# The Feedback Intervention Trial (FIT) — Improving Hand-Hygiene Compliance in UK Healthcare Workers: A Stepped Wedge Cluster Randomised Controlled Trial

**DOI:** 10.1371/journal.pone.0041617

**Published:** 2012-10-23

**Authors:** Christopher Fuller, Susan Michie, Joanne Savage, John McAteer, Sarah Besser, Andre Charlett, Andrew Hayward, Barry D. Cookson, Ben S. Cooper, Georgia Duckworth, Annette Jeanes, Jenny Roberts, Louise Teare, Sheldon Stone

**Affiliations:** 1 Royal Free Campus, University College London Medical School, University College, London, United Kingdom; 2 University College London, London, United Kingdom; 3 Health Protection Agency, London, United Kingdom; 4 University College London Hospitals, London, United Kingdom; 5 London School of Hygiene and Tropical Medicine, London, United Kingdom; 6 Mid-Essex NHS Trust, Chelmsford, United Kingdom; University of Hong Kong, Hong Kong

## Abstract

**Introduction:**

Achieving a sustained improvement in hand-hygiene compliance is the WHO’s first global patient safety challenge. There is no RCT evidence showing how to do this. Systematic reviews suggest feedback is most effective and call for long term well designed RCTs, applying behavioural theory to intervention design to optimise effectiveness.

**Methods:**

Three year stepped wedge cluster RCT of a feedback intervention testing hypothesis that the intervention was more effective than routine practice in 16 English/Welsh Hospitals (16 Intensive Therapy Units [ITU]; 44 Acute Care of the Elderly [ACE] wards) routinely implementing a national cleanyourhands campaign). Intervention-based on Goal & Control theories. Repeating 4 week cycle (20 mins/week) of observation, feedback and personalised action planning, recorded on forms. Computer-generated stepwise entry of all hospitals to intervention. Hospitals aware only of own allocation. Primary outcome: direct blinded hand hygiene compliance (%).

**Results:**

All 16 trusts (60 wards) randomised, 33 wards implemented intervention (11 ITU, 22 ACE). Mixed effects regression analysis (all wards) accounting for confounders, temporal trends, ward type and fidelity to intervention (forms/month used).

**Intention to Treat Analysis:**

Estimated odds ratio (OR) for hand hygiene compliance rose post randomisation (1.44; 95% CI 1.18, 1.76;p<0.001) in ITUs but not ACE wards, equivalent to 7–9% absolute increase in compliance.

**Per-Protocol Analysis for Implementing Wards:**

OR for compliance rose for both ACE (1.67 [1.28–2.22]; p<0.001) & ITUs (2.09 [1.55–2.81];p<0.001) equating to absolute increases of 10–13% and 13–18% respectively. Fidelity to intervention closely related to compliance on ITUs (OR 1.12 [1.04, 1.20];p = 0.003 per completed form) but not ACE wards.

**Conclusion:**

Despite difficulties in implementation, intention-to-treat, per-protocol and fidelity to intervention, analyses showed an intervention coupling feedback to personalised action planning produced moderate but significant sustained improvements in hand-hygiene compliance, in wards implementing a national hand-hygiene campaign. Further implementation studies are needed to maximise the intervention’s effect in different settings.

**Trial Registration:**

Controlled-Trials.com ISRCTN65246961

## Introduction

Controlled trials show [1]–[3] that hand-hygiene significantly reduces spread of infection. However, hand-hygiene compliance amongst healthcare workers remains poor, with levels of 25–40% being common [4]–[6].

Sustained improvements in hand-hygiene are key to the World Health Organisation’s strategy to reduce health-care associated infection [7]–[9]. To that end, many countries have introduced hygiene campaigns [10], [11] but there is no randomised controlled trial evidence showing which intervention improves hospital healthcare workers’ hand-hygiene compliance. Systematic reviews of short-term non-randomised studies [12], [13] suggest that feedback may be the most successful intervention. There is substantial evidence from systematic reviews of randomised controlled trials, that feedback significantly improves healthcare workers’ compliance with other evidence-based guidelines [14], [15] although the improvement is modest, possibly due to the absence of behavioural theory to optimise intervention design [15], [16]. Taken together, these reviews call for well-designed, long-term trials of a feedback intervention to improve hand-hygiene compliance, designed using behavioural theory.

We performed such a trial on wards already implementing a national hand-hygiene campaign [4], [11], [17], testing the hypothesis that a behaviourally designed feedback intervention would produce significant sustained improvements in hand-hygiene compliance compared to routine practice.

## Methods

### Ethics Statement

Ethical approval was received from the Multi-centre Research Ethics Committee (Scotland B) (05/MRE10/2). Hospitals and wards were assigned confidential ID codes. Ward managers, infection control nurses and ward co-ordinators gave written consent.

**Figure 1:**
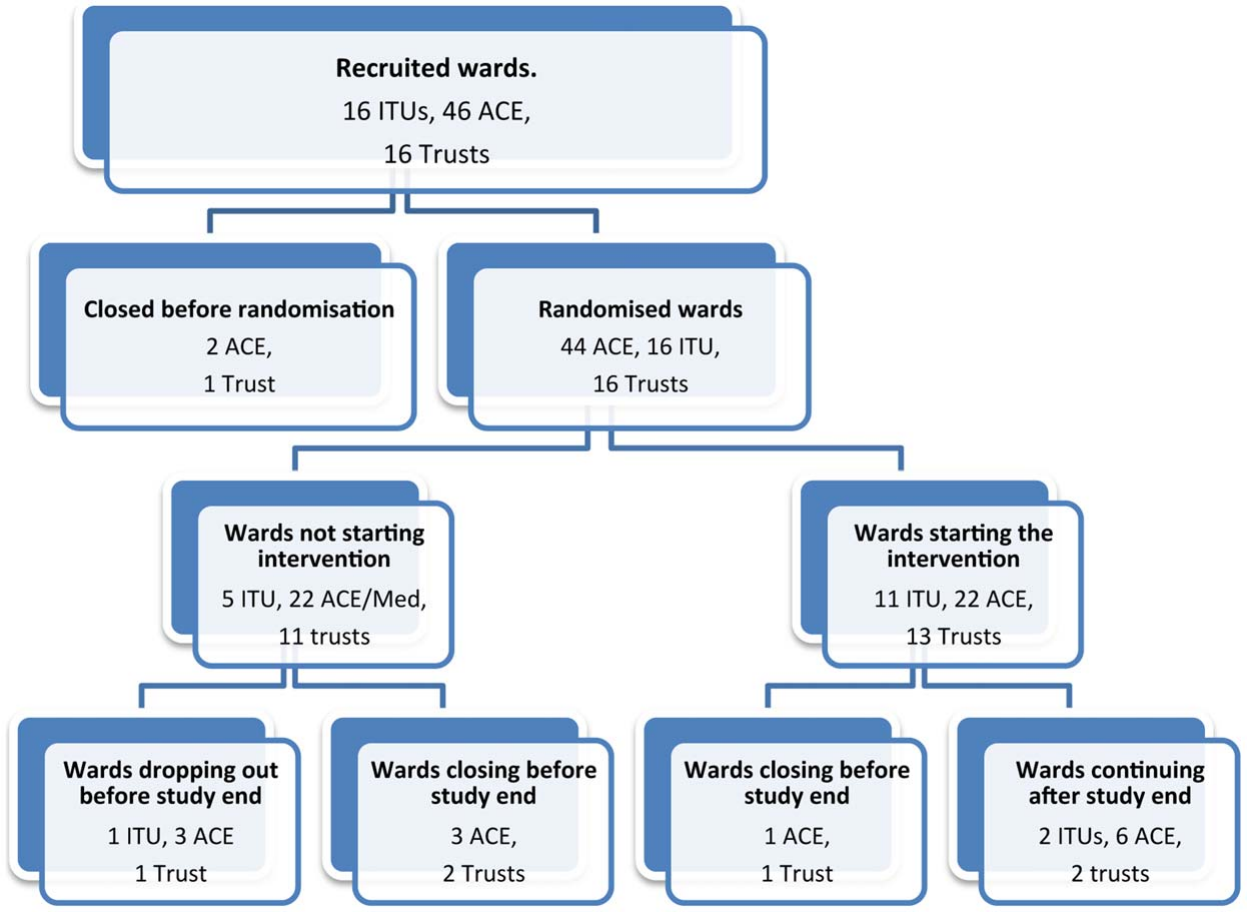
Flowchart showing study recruitment and attrition.

### Trial Design

A stepped wedge cluster randomised controlled design [18] was chosen following piloting to facilitate roll out of the intervention, ensure equity, and prevent contamination and disappointment effects in hospitals not randomised to the intervention.

**Figure 2:**
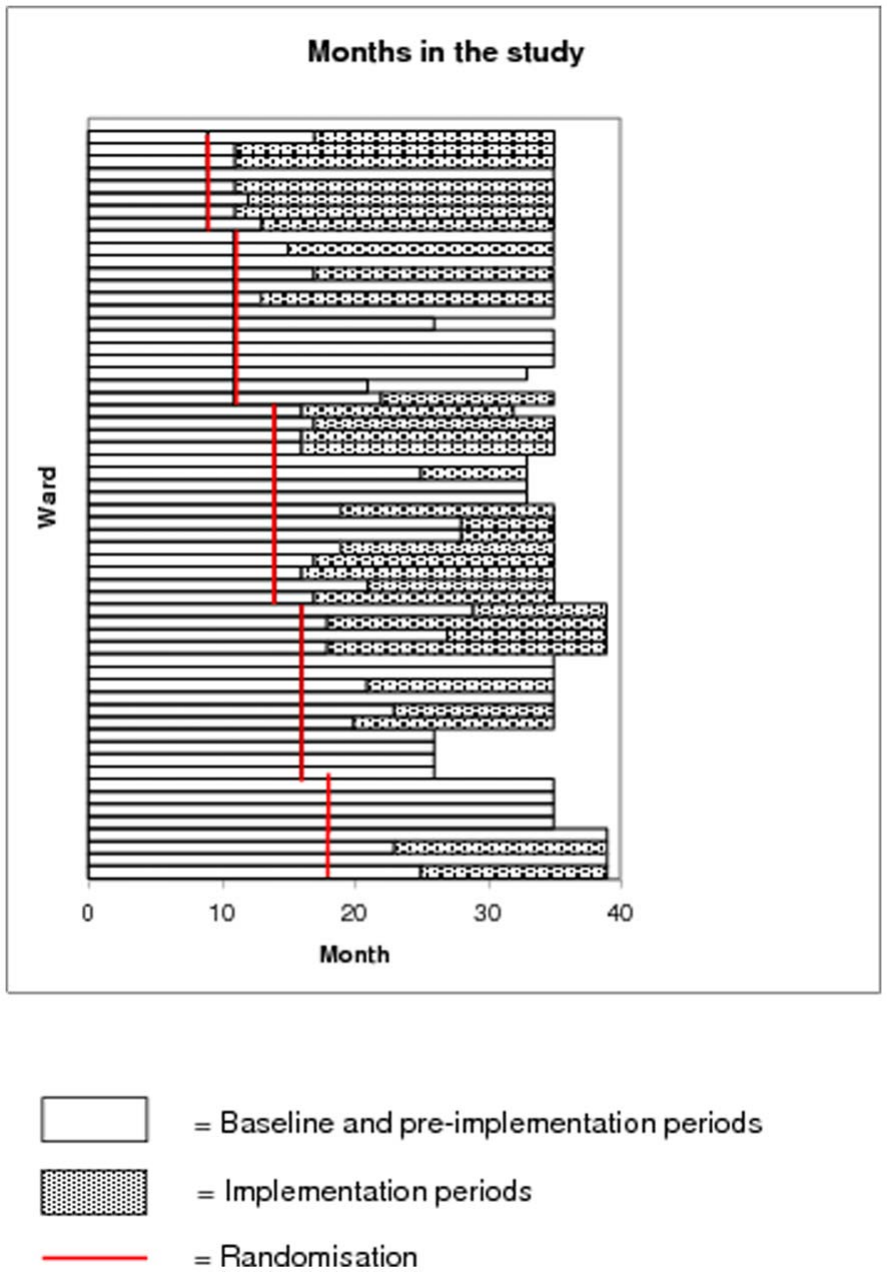
Timeline for randomisation and implementation.

### Participants and Setting

Sixty wards (44 acute care of the elderly or general medical wards [ACE] and 16 intensive therapy units [ITU]) in 16 acute hospitals across England and Wales (14 general acute and 2 teaching hospitals) participated in the trial, as settings known generally to have high levels of healthcare associated infection [19], [20].

**Table 1:** Estimated odds ratios (95% CI) of hand hygiene compliance for the intervention allowing for effect modification by type of ward (intention-to-treat).

Factor	Estimated odds ratio	95% CI	P value
ACE			
Before randomisation	Reference		
After randomisation	1.06	0.87 to 1.27	0.5
ITU			
Before randomisation	Reference		
After randomisation	1.44	1.18 to 1.76	<0.001

**Figure 3:**
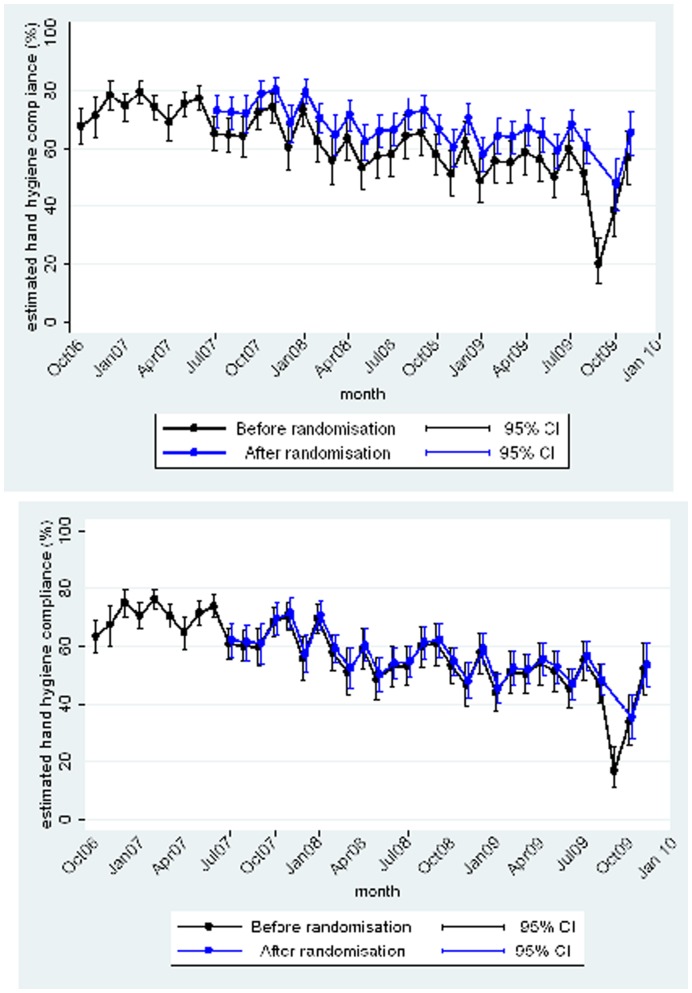
Hand-hygiene compliance in ITUs (upper panel) and ACE wards (lower panel): Intention-to-treat analysis.

### Eligibility Criteria

Hospitals were eligible if, after three or four recruitment visits (the recruitment process is described elsewhere [21] they wished to carry out the trial on the ITU and two or three ACE wards, and were implementing the national clean***your***hands campaign as part of routine practice. This pragmatically designed campaign [4.11] was successfully rolled out to all acute hospitals in England and Wales between December 2004 and June 2005 [17], [21]. It comprised bedside placement of alcohol hand-rub, posters and patient empowerment materials encouraging healthcare workers to clean their hands, plus audit and feedback of hand-hygiene compliance at least once every 6 months.

**Table 2:** Estimated odds ratios (95% CI) for the intervention allowing for effect modification by type of ward in a model excluding the potential confounders (per-protocol analysis).

Factor	Estimated odds ratio	95% CI	P value
ACE			
Before randomisation	Reference		
After randomisation before implementation	1.39	1.08 to 1.80	0.01
After implementation	1.67	1.26 to 2.22	<0.001
ITU			
Before randomisation	Reference		
After randomisation before implementation	1.70	1.26 to 2.30	<0.001
After implementation	2.09	1.55 to 2.81	<0.001

**Figure 4:**
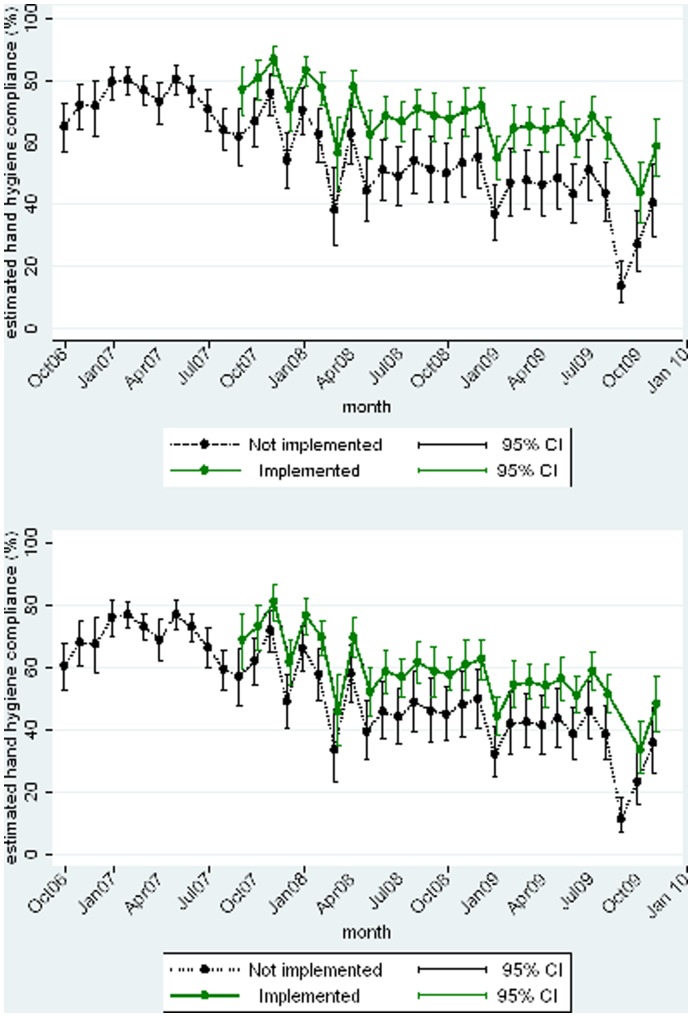
Hand-hygiene compliance in ITUs (upper panel) and ACE wards (lower panel): Per-protocol analysis.

### Intervention

The development of the intervention, using an appropriate behavioural theoretical framework [16] and the MRC framework for complex interventions [22], is described elsewhere [21]–[25], and included an exploratory trial [23] on 7 wards in 3 hospitals (none of which participated in the definitive trial).

**Table 3:** Estimated odds ratios (95% CI) for hand hygiene compliance on ITUs for 0, 1, 2, 3, or 4 forms returned in any one month compared to the compliance prior to randomisation.

Factor	Estimated odds ratio	95% CI	P value
ITU			
After implementation no forms returned	1.83	1.33 to 2.50	<0.001
After implementation one form returned	2.02	1.50 to 2.72	<0.001
After implementation two forms returned	2.23	1.65 to 3.02	<0.001
After implementation three forms returned	2.46	1.78 to 3.40	<0.001
After implementation > = four forms returned	2.71	1.90 to 3.88	<0.001

The Feedback Intervention was designed using Goal-setting [26], Control [27] and Operant Learning [28] theories. The first two conceptualise behaviour as goal-driven and feedback-controlled, with goal-setting and action planning augmenting the effect of feedback. The intervention component based on Operant Learning Theory provided a reinforcement component by associating performance of the target behaviours with reward to increase the frequency of the desired behaviour.

**Table 4:** Estimated relative change (95% CI) in liquid soap procurement by type of ward (intention-to-treat analysis).

Ward	Estimated relative change (95% CI)
ACE	1.133 (0.987 to 1.300) p = 0.08
ITU	1.314 (1.114 to 1.548) p = 0.003

The intervention was carried out by an allocated “ward coordinator”, a junior ward sister or infection control link nurse, and involved a repeating four-week cycle.

#### Week 1

Hand-hygiene observation of an individual Nurse/Health Care Assistant for 20 minutes. Immediate feedback was given after the period of observation, and, for instances of non-compliance with hand-hygiene, the person observed was helped formulate an action plan to improve behaviour. For example, when a healthcare worker didn’t clean hands after touching patient equipment but not the patient, the action was set as *“X will use alcohol hand-rub even if only touching patient equipment”.*

Observation was discreet, as described elsewhere [25]. If compliance was 100%, the staff member was praised and given a certificate that was filed for use in annual professional development appraisal. If there were two or more instances of poor compliance during observation, the staff member was observed at some point within the subsequent month. The aim was to observe every member of staff at least once a year.

**Table 5:** Estimated relative change in soap procurement on ITUs for 0, 1, 2, 3, or 4 forms returned in any one month compared to the compliance prior to randomisation.

ITU			
After implementation no forms returned	1.10	0.85 to 1.41	0.5
After implementation one form returned	1.22	0.98 to 1.54	0.08
After implementation two forms returned	1.37	1.09 to 1.72	0.007
After implementation three forms returned	1.53	1.19 to 1.96	0.001
After implementation > = four forms returned	1.71	1.28 to 2.28	<0.001

#### Week 2

As for week one except that a “non-nurse” (doctor or other healthcare professional) was observed.

#### Week 3

Hand-hygiene observation of a ward area for 20 minutes, recording the hand-hygiene behaviour of all healthcare workers entering that area (group compliance). Poor practice was documented but feedback was not given at the time.

#### Week 4

The week 3 observations (group compliance) were fed back and action plans formulated at a ward meeting. For example, when student nurse practice was observed to be poor, the following action plan was set. *“All student nurse assessors to take student nurses through hand-hygiene practice on arrival on ward”.*

#### Fidelity to intervention

Ward co-ordinators were asked to fill out a form to record, observations, feedback, goals and action plans (www.idrn.org/nosec.php) each time an observation and/or feedback session took place and to return them to the study team. The number of forms returned each month was used as a proxy measure of fidelity to intervention.

#### Training the ward co-ordinators

Ward co-ordinators were trained in hand-hygiene observation [22] and how to provide feedback, help healthcare workers to set their own hand-hygiene goals and make action plans. Training comprised discussion of the training materials and a series of structured exercises, delivered by study personnel and usually completed in 1 to 1 ½ hours (www.idrn.org/nosec.php). In total 62 training visits were made to hospitals. These could be difficult to organise. Representatives from 11 wards (7 hospitals) never attended training. Initial visits were followed up 6–8 weeks later and further training was given as requested (36 wards) up to six months after starting the intervention.

#### Outcomes

Data were collected from 1^st^ October 2006 to 31st December 2009.

#### Primary outcomes

Hand-hygiene compliance was measured by covert direct observation by an observer blinded as to ward allocation or randomisation to the intervention. The adequacy of blinding was tested and confirmed [29]. Observation periods were for one hour, every 6 weeks, using the Hand Hygiene Observation Tool [25], which has proven reliability and sensitivity to change. Compliance was expressed as a percentage of the hand-hygiene moments that were associated with observed hand-hygiene behaviour (use of alcohol hand-rub or soap).

#### Secondary outcomes

Monthly soap and alcohol hand-rub procurement data (litres per bed day) were collected as a proxy measure of hand-hygiene compliance for each ward, as this reflects 24-hour, seven days a week use, and is neither subject to observer bias or reactive effects. Data were collected from hospital supplies departments or NHS Supply Chain.

#### Tertiary outcomes

Anonymised confidential MRSA prevalence swabs were to be collected quarterly but, despite receiving ethical approval, only 12 wards (three hospitals) agreed to this and therefore it had to be abandoned. Information on other healthcare associated infection outcomes collected, but for which the study was underpowered to detect significant change, is reported elsewhere [21].

#### Denominator

*Bed-days-* ward bed-days per month were recorded to act as a denominator for alcohol hand-rub and soap procurement data.

#### Potential confounding factors

Staffing levels – numbers of registered nurses, healthcare assistants and bank staff. These data were only collected for days on which hand-hygiene observations were undertaken by the study researchers as potential residual confounders affecting the intervention.

#### Sample size

The methods for sample size calculations are fully described elsewhere [21] and comprise a simulation approach [30], parameterised by exploratory trial observations [23] on one ITU and 3 ACE wards. A linear “mixed” model was fitted to the simulated compliance data which gave a stepped wedge trial of 36 months duration and six-weekly hand-hygiene observations 79% power to detect differences in hand-hygiene compliance of 7% or greater for 16 hospitals, and 89% power to detect differences of 8% or more.

#### Randomisation

After an initial baseline period hospitals were randomised into the intervention at two monthly intervals (Figure 1).

#### Sequence generation

Hospitals were allocated a number between 1 and 16. Numbers were randomly sorted using the Research Randomiser website. (http://www.randomizer.org/form.htm). Hospitals entered into the intervention in this order in blocks of 2 to 4, at five predefined time-points. The first two hospitals were randomised to start in month 10 (July 2007) and the final two in month 19 (April 2008). All study wards within the hospital were allocated to start the intervention concurrently.

#### Allocation concealment mechanism

Infection control teams and ward managers were informed of their own hospital’s allocation in May 2007. Only the research team knew the allocation of all hospitals.

#### Statistical methods

The binomial proportion of the number of compliant hand-hygiene opportunities in the total number of hand-hygiene opportunities at each of the ward visits during the study was the primary outcome variable. This was analysed using mixed-effect logistic regression, allowing for dependencies of observations made within hospitals and wards by incorporating these as hierarchical random effects. To account for general secular temporal trends in compliance over the study, compliance was able systematically to vary from month-to-month by its inclusion as a categorical predictor variable. For the “intention-to-treat” analysis an indicator of whether an observation occurred pre- or post-randomisation was included in the regression model. To allow for delays in implementation a separate “per protocol” analysis was performed with the observations now placed into one of the three categories: “pre-randomisation”, “post-randomisation but pre-implementation” and “post-implementation”, in case behaviour altered once randomised wards knew they were to receive the intervention. Additional technical information is provided in Text S1.

The number of registered nurses, healthcare assistants and bank staff, and the ratio of actual to expected staff numbers were fitted as covariates to control for any residual confounding that may arise from unbalanced randomisation at the group level. The type of ward (ACE or ITU) was considered an effect modifier, with the interaction between this and the intervention variable included in models. Fidelity to intervention was fitted as a covariate, and its interaction with ward type assessed.

Estimated odds ratios (95% CI) were obtained for hand-hygiene compliance, comparing post-randomisation and post-implementation with pre-randomisation compliance, allowing for effect modification by ward type.

A linear mixed regression analysis was performed for the secondary outcomes. The monthly volume of alcohol hand-rub/liquid soap procured was smoothed to allow for bulk orders, divided by the number of bed days and a logarithmic transformation was applied.

#### Protocol

There was a predefined protocol (www.idrn.org/nosec.php) which was followed except for three violations. Firstly, it was not possible to perform the MRSA prevalence screening (see above). Secondly, a questionnaire measuring ward culture was filled out by so few nurses that this was dropped from the protocol. Thirdly, delayed Research and Development registration shortened the baseline pre-randomisation phase from twelve months to nine in the first hospitals randomised to the intervention. Both the protocol and supporting CONSORT checklist are available as supporting information: see Protocol S1 and Checklist S1).

## Results

### Participant Flow

The trial start and finish dates were pre-specified as 1^st^ October 2006–30^th^ September 2009. The flow diagram (Figure 1) shows there were 60 study wards in the 16 randomised hospitals, of which 33 (22 ACE and 11 ITU) in 13 hospitals went on to implement the intervention, with a mean (SD) delay in implementation of 5 (4) months (Figure 2) and a mean (SD) duration of implementation of 12 (7) months. Eight wards began implementation very late, and for these the end of the trial was extended to December 31^st^ 2009 to ensure that they had a year of data collection post-implementation.

### Numbers Analysed

For the primary outcome, intention-to-treat analysis was conducted for the 60 wards randomised into the intervention, and per-protocol analysis was performed for the 33 implementing wards.

For the secondary outcomes, adequate soap data were available for 28 wards, 16 of which implemented the intervention (4 ITUs and 12 ACE) and 12 of which did not (3 ITUs and 9 ACE). Adequate alcohol hand-rub data was available for 37 wards, 23 of which implemented the intervention (10 ITUs and 13 ACE) and 14 of which did not implement the intervention (14 ACE wards). Intention-to-treat analysis was carried out for all 28 wards with adequate soap data and for all 37 wards with adequate AHR data. Per-protocol analysis was carried out for all 16 implementing wards with adequate soap data and for all 23 implementing wards with adequate AHR data. Only 15 wards had adequate soap and alcohol hand-rub data together (2 ITUs and 13 ACE), of which only 9 wards (ACE) implemented the intervention.

If fidelity to intervention had been 100%, this should have generated four forms returned every four weeks from each ward i.e. a total of 4968. The total number of forms returned was 974 (19.6%), range 0–69 per ward, representing 33.1% of the 2948 forms expected from the 33 implementing wards. Data were available from all wards as to whether 0,1,2,3 or 4 forms had been returned each month of the study.

### Primary Outcome (Hand-hygiene Compliance)

The initial intention-to-treat analysis showed no effect of any potential confounders, which were then excluded from the analysis. There was a highly significant effect of the intervention in ITUs but not on ACE wards (Table 1). Although hand-hygiene compliance gradually fell during the trial, the increased odds of hand-hygiene compliance in ITUs equated to an absolute increase of 9% when the hand-hygiene compliance without the intervention was 50% and to an increase of 7% when hand-hygiene compliance without the intervention was 70% (Figure 3). In the ACE wards, where the intervention had no significant effect, this equated to an absolute increase in hand-hygiene compliance of only 1%. Fluctuations seen in the last three months in Figure 3 reflect the fact that these data points are based only on the 8 wards who implemented very late and for whom the end of the trial was extended (see above). Excluding these data points, it appears that the intervention maintained compliance on ITUs at 61% by the end of the study whereas without the intervention it fell from 63% to 52%.

Per-protocol analysis in implementing wards (Table 2) showed a highly significant increase in the estimated odds of hand-hygiene compliance in both types of ward. This equated to an absolute increase in hand-hygiene compliance of 13% in ACE wards when hand-hygiene compliance without the intervention was 50%, and of 10% when hand-hygiene compliance without the intervention was 70%. For ITUs this equated to an absolute increase of 18% when hand-hygiene compliance without the intervention was 50% and of 13% when hand-hygiene compliance without the intervention was 70% (Figure 4). Fluctuations seen in the last three months again reflect the fact that these data points come from only eight wards. Excluding these data points it appears that without the intervention compliance fell on ITUs from 61% to 43% by the end of the study, whereas on the implementing wards compliance was maintained at 61% by the end. For ACE wards, whereas compliance fell from 58% to 39% by the end of the study on non implementing wards, the intervention appeared to reduce this fall to 52% on implementing wards.

There was no significant difference in the odds of hand-hygiene compliance pre-randomisation between implementers and non-implementers for ITUs (0.82 [0.61, 1.11]; p = 0.2) or for ACE wards (1.12[0.93, 1.35]; p = 0.2).

Table 3 shows a significant effect of fidelity to intervention on ITUs, with strong evidence of an increase in hand-hygiene compliance. The estimated odds ratio for an increase in hand-hygiene compliance for each returned form is 1.103 (95% CI 1.026 to 1.188, p = 0.008). There was no such effect seen in ACE wards, with the estimated odds ratio for each returned form being 0.998 (95% CI 0.948 to 1.050, p = 0.9).

#### Secondary outcomes (soap and alcohol hand-rub procurement)

Table 4 summarises the intention-to-treat analysis and shows that liquid soap procurement increased significantly by over 30% post-randomisation in ITUs, with a non-significant trend towards increasing procurement in ACE wards of 13%. There was no evidence of a rise in alcohol hand-rub procurement with the estimated relative change (95% CI) post-randomisation of 1.064 (0.933 to 1.214); p = 0.4 in ITUs and 1.027 (0.919–1.148); p = 0.6 in ACE wards.

The per-protocol analysis also showed a 30% rise in soap procurement in ITUs (95% C.I.) 1.3 [1.03–1.63]), but not in ACE wards (1.02 [0.84–1.25]). However, this result is based on only four implementing ITUs with adequate soap data. For these wards, Table 5 shows a significant effect of fidelity to intervention, the estimated relative change per form returned being 1.118 (95% CI 1.039 to 1.202, p = 0.003). There was no such effect in the 12 implementing ACE wards with adequate soap data, the estimated relative change per form returned being 0.973 (95% CI 0.937 to 1.010, p = 0.16).

Per-protocol analysis showed no increase in alcohol hand-rub procurement for the wards with the estimated relative change post implementation being 1.183 (0.989 to 1.416) for ACE wards and 1.098 (0.904 to 1.333) for ITUs. There was no evidence of an effect of fidelity to intervention with the estimated relative change per form returned being 1.01 (95% CI 0.98 to 1.05, p = 0.5), and 1.02 (95% CI 0.96 to 1.07, p = 0.5), in the ACE and ITU wards respectively.

## Discussion

The principal findings of this trial were that a feedback intervention, designed using behavioural theory, produced a moderate but significant sustained improvement in hand-hygiene compliance on both intention-to-treat and per-protocol analyses, on wards whose routine practice included implementation of the pragmatically designed national hand-hygiene campaign. This confirmed the original trial hypothesis, despite difficulties in implementation and a downwards temporal trend in hand-hygiene compliance over the study period. The effect was stronger on ITUs, where it was easier to implement and where its effectiveness increased with fidelity to intervention. The effect of the intervention on implementing wards equated to an absolute difference in hand-hygiene compliance of 13–18% on ITUs, and of 10–13% on ACE wards. This was relatively constant over time, consistent with a sustained effect.

The principal strength of the study is that it met the requirements of systematic reviews calling for large well-designed long-term trials of hand-hygiene interventions [12], [13] which apply behavioural theory to intervention design [15], [31], [32]. The stepped wedge design increases power as wards act as their own control and the extended duration allows assessment of sustainability [18]. The ability to control for temporal trends allows effectiveness to be assessed even against a background of a successful [17] national hand-hygiene campaign.

The study’s main limitation was that the intervention was more difficult to implement than in the exploratory trial [23]. Such difficulties are well documented [33] in healthcare settings, but may also reflect changes in the National Health Service, including having to compete with other quality improvement initiatives. Cross-sectional interviews in the exploratory trial suggested that implementation might increase if the intervention were an integral part of a hospital’s audit programme, carried out by infection control or ward staff with general responsibilities for assessment and appraisal, with more than one co-ordinator per ward, each having protected time for delivering the intervention [23].

A second limitation was that ward implementers, once trained, neither had their training repeated nor their performance monitored. This might have reduced the effect of the intervention, and been partly responsible for the gradual decline in compliance seen during the study. This gradual fall might also reflect a possible wearing off of the national campaign over its final year (January-December 2008), or generic changes in working practices and pressures in the health service. Although this fall suggests some caution should be exercised in interpreting the effect of the intervention, the nature of the study design can cope with temporal trends which were allowed for in all analyses.

A final limitation was the difficulty collecting secondary and tertiary outcome data. The reluctance of ward staff to perform MRSA prevalence screening meant that no conclusions can be drawn regarding the effect of the intervention on healthcare associated infection. Collection of alcohol hand-rub and soap procurement was not a problem in the exploratory trial, but arose from lack of ward-level requisition or recording points in individual hospitals. Despite this, the effect of the intervention on soap procurement mirrored that on directly observed compliance for ITUs and provides further support for its efficacy.

Our results are consistent with systematic reviews [14], [15] of 61 randomised controlled trials of the effectiveness of audit and feedback on healthcare practices other than hand-hygiene. These report a significant effect of about the same size (adjusted odds ratio of compliance with desired practice 1.43 [1.28, 1.61]) that, as in our study, increased with increasing intensity of feedback, and lower baseline compliance.

Comparison with other hand-hygiene feedback intervention studies [34]–[42] included in systematic reviews [12], [13] is difficult because this is the only long term randomised controlled trial, and the only one coupling feedback to personalised goal setting and action planning. No other study compares their intervention with a baseline that includes another specific hand-hygiene intervention, whereas in our study standard practice included implementation of a national hand-hygiene campaign.

This gives the trial’s findings extra relevance, as subsequent studies [17], [21], [43] have shown that the campaign was widely implemented and successful. Although we did not routinely collect data on implementation of the campaign from infection control and ward staff, in case this acted as a prompt to alter “routine practice”, data on near-patient placement of alcohol hand-rub, the key component of the campaign, was routinely collected in the hand-hygiene observation tool [22]. This showed that this component had been implemented on all wards throughout the study. The World Health Organisation’s SAVE LIVES initiative promotes a hand-hygiene intervention very similar to the English and Welsh hand-hygiene campaign [8]. Our study suggests that our intervention may improve hand-hygiene in such settings and could be the next step in hand-hygiene improvement after a hospital has adopted the SAVE LIVES intervention.

It would clearly be premature to recommend routine clinical use of our intervention as it is hard to comment on the generalisation of our results to settings other than ITUs and ACE wards, or to health services or countries with no sustained national hand-hygiene campaign. The post-randomisation pre-implementation rise in the odds of compliance on implementing wards may indicate that there were characteristics of those wards that eventually facilitated implementation. Those characteristics do not appear to include better baseline hand-hygiene compliance as there was no difference in the odds of pre-randomisation hand-hygiene compliance in implementing and non-implementing wards. Possible reasons for the greater implementation in ITUs are entirely speculative but include a higher degree of training and specialisation and a larger staffing pool from which to recruit ward co-ordinators. A further implementation study in a variety of settings is required, with the performance of ward co-ordinators monitored, and cost-effectiveness models developed, before the intervention can be offered routinely in acute hospital settings. This needs to be informed by further research to identify what each component of the intervention contributed to its effect. Nonetheless, hospitals keen to improve their hand-hygiene compliance could consider employing this intervention, with the same cycle and behavioural principles of feedback, to supplement their current audit and appraisal systems.

In conclusion, the current study has shown that a feedback intervention informed by behavioural science results in moderate significant and sustained increases in hand-hygiene compliance and soap procurement on wards already implementing a national hand-hygiene campaign as part of routine practice. The effect increases with fidelity to intervention. The intervention proved harder to implement than anticipated, and further implementation studies are required. Although audit and feedback is often suggested as a useful tool for hand-hygiene improvement [5], [34], [44], this study puts its use on a firmer footing than previous non-randomised studies, providing the strongest evidence yet that this is an effective technique, when coupled with a repeating cycle of personalised goal-setting and action planning.

## Supporting Information

Checklist S1 **CONSORT checklist for FIT.**
(DOC)

Protocol S1 **Feedback Intervention Trial Protocol.**
(DOC)

Text S1 **Model formula and general statistical approach to stepped wedge trials.**
(DOC)
