# Supplementary material for: The Feedback Intervention Trial (FIT) — Improving Hand-Hygiene Compliance in UK Healthcare Workers: A Stepped Wedge Cluster Randomised Controlled Trial
Source: PLoS One. 2012 Oct 23;7(10):e41617. doi: 10.1371/journal.pone.0041617 (PMC3479093; doi:10.1371/journal.pone.0041617)
Supplement: Protocol S1 — Feedback Intervention Trial Protocol. (DOC) [file pone.0041617.s002.doc]

Feedback Intervention Trial Protocol

**TITLE:** The Feedback Intervention Trial (FIT): a stepped wedge cluster randomised controlled trial of the effect of a feedback intervention on hand hygiene compliance in healthcare workers on intensive therapy units (ITUs) and Acute Care of the Elderly/General medical (ACE/GM) wards.

***Hypothesis:*** The null hypothesis was that the feedback intervention would have no effect on hand hygiene compliance compared to standard practice, which included routine use of the pragmatically designed national clean***your***hands campaign (1).

***Design*:** stepped wedge cluster randomised controlled trial

***Participants*:** 16 trusts in England and Wales with at least 1 ITU and 2 ACE/GM wards per trust.

***Eligibility criteria:*** the ward managers and Infection control nurses will have agreed to the trial, appointed a ward co-ordinator and given consent (05/MREC10/2, Scotland B, multi-centre research ethics committee). The hospital and wards will have implemented the cleanyourhands campaign as part of routine hand hygiene practice, and will not adopt any other hand hygiene interventions during the time of the trial. Hospitals will have at least one ICN per 300 beds. Of the 16 hospitals, 2 or 3 should be teaching hospitals to provide a representative mixture of hospitals in England and Wales.

***Intervention:*** based on goal setting and control theories, the intervention consists of a repeated four week audit cycle of observation of individual HCWs and groups of HCWs using the behaviour change techniques of observation, feedback, agreement of goal setting and action plans, and reward. Intervention will be implemented by a ward co-ordinator, either a ward manager or sister, an infection control link nurse, or an infection control nurse, and will be delivered in the manner described in the training materials developed for the trial (www.idrn.org/nosec.php).

In week one an individual nurse will be observed for 20 minutes, using a standard methodology, the hand hygiene observation tool) (www.idrn/nosec.php) developed by the research team and based on the Geneva tool for observing hand hygiene compliance (2). Any lapses in compliance will be feedback immediately to the nurse or health care assistant, and a discussion will help them agree on goals and action plans to improve their compliance in future. If their compliance is 100% they will receive a certificate to be placed in the CPD folders. If there are more than two instances of poor compliance, they will be reassessed on a subsequent occasion.

In week 2: a non nurse (doctor or physiotherapist for example) will be observed in the same way.

In week three: a group of HCWs will be observed and their compliance and any lapses noted. Pbservations will be documented on the form designed for this purpose. Feedback however will not be given at that point.

In week 4: ward co-coordinators will feedback group compliance to the ward team e.g. at the monthly team meeting) and agree goals and action plans. The observations, feedback, goals and action plans will be recorded as described above on a specific form.

All forms from weeks 1, 2, 3 and 4 will be returned to the research team and the number filled in per month will act as a proxy measure of fidelity to intervention

***Randomisation:*** A stepped wedge computer generated randomisation of hospitals into the intervention ensure entry of four hospitals at a time into the intervention at four predetermined intervals four months apart, after an initial baseline year (months 0-12). Hospitals will be entered into the intervention at months 13, 17, 21 and 25. Hospitals will only be aware of their own time of randomisation into the intervention but only the research fellow and research assistant co-ordinating the trial will be aware of other hospitals times of randomisation.

***Outcomes:***

**Primary** - hand hygiene compliance measured by observers blinded to the hospital allocation, using the developed methodology ([www.idrn/nosec.php](http://www.idrn/nosec.php)). This records hand hygiene opportunities and associated hand hygiene behaviours (use of soap, alcohol hand rub, or no action). Observations will be carried out for an hour at a time on each ward, every 6 weeks. Although observations will take place in the morning and early afternoon, observations taken for any one ward will always be taken at the same time of day as the first observations made on that ward in order to control for variation in ward activities dependent on the time of day. Blinding of the observer to ward allocation will be checked.

**Secondary** : ward based procurement of alcohol hand rub and soap in Litres per bed day per month.

**Tertiary :** the following infection outcomes will be examined

1. MRSA prevalence swabbing will be carried out three times a year in each ward which will be the main infectious outcome.
2. Ward based healthcare associated infection monthly data routinely collected as part of national mandatory reporting of MRSA Bacteraemia, MSSA bacteraemia, and Clostridium difficile infection as cases per 10,000 days.
3. The wards own routine surveillance data for new MRSA acquisitions (usually defined locally as new MRSA detection less than 48 hours after admission) will also be collected.
4. A proxy marker of total MRSA infection consisting of the daily defined doses per 10,000 bed days (3) of the common anti MRSA antibiotics such as teicoplanin, vancomycin IV and doxycycline) will also be collected.

***Sample size-power calculations:*** A simulation approach based on hand hygiene compliance data from a pilot study developing the intervention and data collection systems was used to provide an estimate of power for a stepped wedge design of total duration 36 months, six weekly hand hygiene observations of compliance in each ward, with one ITU and two ACE wards at each trust. Linear mixed effects modelling was fitted to the simulated hand compliance data, and 1000 simulations were performed for each combination of intervention effect from 0% to 12% increase in compliance in steps of 1%, and number of participating trusts 10, 16, and 20. This gave the trial 79.2% power at the 5 % significance level to detect differences in hand hygiene compliance of 7% or greater for 16 trusts, and 88.8% power to detect differences of 8% or more.

***Confounders:*** (i) data on staffing levels and skill mix will be collected for each ward for the day of hand hygiene observations by the hand hygiene observer.

(ii) Monthly data on average bed occupancy, average length of stay, number of female admissions and source of admission (care home or nursing home) will be collected if routinely available by research workers liaising with hospital information specialists at each hospital.

(iii) Ward level defined daily doses of ciprofloxacin and cephalosporins, as these might affect levels of MRSA Bacteraemia and Clostridium difficile infection will be collected by the research team liaising with hospital pharmacy departments.

(v) Every 6 months ward staff will be invited to fill out an organisational climate questionnaire to assess ward culture.

***Confidentiality:*** Each trust, hospital, and ward will be assigned a confidential ID code and any patient data will be collected in anonymised aggregate form.

***Analysis:*** Generalised linear mixed (multilevel) models will also be used to analyse the longitudinal data generated above with the primary comparison being between pre randomisation and post randomisation hand hygiene compliance. This will be done on an intention to treat and per protocol basis. The model will take into account the hierarchical nature of the design (measurements nested within hospitals, and, if appropriate, hospitals within trusts), autocorrelation, pre-intervention trends, seasonal effects, fidelity to intervention and measured confounding factors. Response functions for modelling the effect of the intervention will allow for both changes in level and trend of outcome measures and for the possibilities of learning curves associated with the campaign and subsequent decays in improvements in HH. Results will be analysed for the trial as a whole and then look for subgroup effects with respect to ITUs and ACE wards as these are two very different ward settings and patient groups.

***Economic analysis:*** A simple cost effectiveness model will of the intervention will be used rather than a full economic evaluation of the intervention. The costs of the intervention will be estimated by using hospital accounts for staffing and consumables (Alcohol hand rub and soap), recording time spent by each ward co-coordinators on the intervention using a diary they will keep for that purpose. Changes in the numbers of patients acquiring an infection, the reduction if any in days spent in hospital and the associated reduction in costs of therapy and investigations will be estimated at full cost, distinguishing between capital and overhead costs and consumables, allowing the cost per bed day and case to be estimated. The outcome will be expressed in both costs saved and bed days saved using hospital management cost data adjusted where necessary to reflect opportunity costs of running each ward. The opportunity costs will also be translated into the number of cases awaiting treatment who could potentially be admitted. Detailed sensitivity analysis would be undertaken to assess the robustness of the model for variations in the cost or resource parameters. The cost-effectiveness of the intervention would expressed by ward or cluster to show the incremental cost-effectiveness of the intervention per case, per bed day and other outcome measures such as MRSA prevalence and will be included along with other outcome measures in the modelling analysis.

.

***References***

1. Pittet D, Hugonnet S, Mourouga P, et al. Effectiveness of

a hospital-wide programme to improve compliance with

hand hygiene. Lancet 2000;356:1307e1312.

1. National Patient Safety Agency 2004. Achieving our aims. Evaluating the results of the pilot Cleanyourhands campaign.

[www.npsa.nhs.uk/EasySiteWeb/GatewayLink.aspx?alId=5924](http://www.npsa.nhs.uk/EasySiteWeb/GatewayLink.aspx?alId=5924)

1. WHO Collaborating Centre for Drug Statistics Methodology, ATC classification index with DDDs 2005. Oslo 2004.

August 2006.
