# Supplementary material for: The Feedback Intervention Trial (FIT) — Improving Hand-Hygiene Compliance in UK Healthcare Workers: A Stepped Wedge Cluster Randomised Controlled Trial
Source: PLoS One. 2012 Oct 23;7(10):e41617. doi: 10.1371/journal.pone.0041617 (PMC3479093; doi:10.1371/journal.pone.0041617)
Supplement: Text S1 — Model formula and general statistical approach to stepped wedge trials. (DOC) [file pone.0041617.s003.doc]

**Text S1**

1. **Model Formula**

“Intention to treat” model

“Per-protocol” model

Where;

*π* denotes estimated proportion of compliance

*β* denotes estimated regression coefficient for the predictor variables

*i* denotes hospital i

*j* denotes ward j

*k* denotes month k

*spec* is an indicator (0=ACE, 1=ITU),

*trt* is an indicator (0=pre randomisation, 1=post randomisation),

*trt*_1_ is an indicator (0=pre randomisation, 1=post randomisation pre implementation),

*trt*_2_ is an indicator (0=pre randomisation, 1=post implementation),

*month* is an factor for sequential month

*u*_i_ is a random effect for hospital (~N(0,σ^2^_h_)

*v*_ij_ is a random effect for wards within (~N(0,σ^2^_w_)

1. **Approach to statistical analysis of stepped wedge trials**

There have been many suggested approaches to the analysis of stepped wedge designs, ranging from repeated application of purely between-unit analysis for each step, to the application of purely within-unit analysis, usually performed by some form of interrupted time series analysis. We have followed the general approach as set out by Hussey and Hughes (Design and analysis of stepped wedge cluster randomized trials. *Contemporary Clinical Trials* 28 (2007) 182–191).

The primary objective of the study is to estimate the effectiveness of the intervention. We agree that the time dimension introduces complexity to this study and this was to some degree expected given the national hand hygiene campaign that occurred prior to the study. However, the time dimension in this study is not of any real interest, in fact, it is a nuisance that has to be taken into account to ensure the effect of the intervention is not unduly biased. The suggestions of using interrupted time series or segmented regression analysis is possible, however, this would require explicit knowledge of the nature of the external initiative to ensure that an unbiased estimate of the effect of the intervention could be obtained. As stated by Hussey and Hughes *“Analyses that rely on within-cluster information only (e.g. paired t-test) provide a valid analysis of the stepped wedge design only if there are no time effects. Otherwise, a within-cluster analysis provides a biased estimate of the treatment effect”.*The complex sequencing of the wards into the study is a reflection of the nature of the stepped wedge design. The generalised linear mixed model (GLMM) used in this analysis provides an estimate of the intervention effect, using both between and within ward effects where the outcome is non-Gaussian. To remove any temporal patterns, both cyclical and secular, a factor for the consecutive month has been incorporated as a fixed effect in the regression model.
